# Isocitrate dehydrogenase 2 contributes to radiation resistance of oesophageal squamous cell carcinoma via regulating mitochondrial function and ROS/pAKT signalling

**DOI:** 10.1038/s41416-020-0852-4

**Published:** 2020-05-05

**Authors:** Xuan Chen, Shichao Zhuo, Wenzhe Xu, Xue Chen, Di Huang, Xiaozheng Sun, Yufeng Cheng

**Affiliations:** 1 Department of Radiation Oncology, Qilu Hospital of Shandong University, 250012 Jinan, Shandong China; 2 Department of Pathology, Xuzhou Central Hospital, 221009 Xuzhou, Jiangsu China; 3 Department of Neurosurgery, Qilu Hospital of Shandong University and Institute of Brain and Brain-Inspired Science, Shandong University, 250012 Jinan, Shandong China

**Keywords:** Oesophageal cancer, Radiotherapy, Tumour biomarkers, Molecular biology

## Abstract

**Background:**

Antioxidase alleviates the accumulation of radiation-induced reactive oxygen species (ROS) and therefore has strong connections with radioresistance. Isocitrate dehydrogenase 2 (IDH2) facilitates the turnover of antioxidase, but its role in radiotherapeutic efficiency in oesophageal squamous cell carcinoma (ESCC) still remains elusive.

**Methods:**

The involvement of IDH2 in radiotherapeutic efficacy in ESCC was investigated in vitro and vivo by IDH2 knockdown. IDH2 expression in biopsy specimens of 141 patients was identified to evaluate its clinical significance.

**Results:**

We found that Kyse510 and Kyse140 cells were more radioresistant and had higher IDH2 expression. In these two cell lines, IDH2 knockdown intensified the radiation-induced ROS overload and oxidative damage on lipid, protein, and nucleic acids. In addition, IDH2 silencing aggravated the radiation-induced mitochondrial dysfunction and cell apoptosis and ultimately promoted radiosensitisation via inhibiting AKT phosphorylation in a ROS-dependent manner. Furthermore, IDH2 depletion facilitated the radiation-induced growth inhibition and cell apoptosis in murine xenografts. Finally, IDH2 expression was correlated with definite chemoradiotherapy (dCRT) efficacy and served as an independent prognostic factor for survival of ESCC patients.

**Conclusions:**

IDH2 plays a key role in the radioresistance of ESCC. Targeting IDH2 could be a promising regimen to improve radiotherapeutic efficiency in ESCC patients.

## Background

Oesophageal squamous cell carcinoma (ESCC) is the most frequent histological type of oesophageal cancer globally.^1,2^ The majority of ESCC patients are diagnosed at a locally advanced stage with long oesophageal lesions and serious invasion and/or enlargement of the lymph nodes,^2^ and large prospective Phase 3 trials have established definite chemoradiotherapy (dCRT) as the standard treatment for those patients.^3,4^ However, the curative effects of radiotherapy differ from those in patients who have an identical malignancy grade and are homogeneously treated. In addition, a large proportion of ESCC patients suffer from recurrence and metastasis even after clinical complete response (cCR). Inherent heterogeneity caused by different gene expression may contribute to that phenomenon.^5,6^ Therefore, exploring predictive biomarkers for radiotherapeutic response should be emphasised.

The isocitrate dehydrogenase (IDH) family are the rate-limiting enzymes in the tricarboxylic acid cycle that reversibly convert isocitrate to α-ketoglutarate, thereby producing NADPH.^7^ IDH2 is localised in mitochondria and critical for maintaining mitochondrial redox homoeostasis.^8–10^ NADPH generated by IDH2 helps to regenerate the reduced glutathione (GSH) and thioredoxin (TXN), which are key antioxidants against reactive oxygen species (ROS).^11^ Inhibiting GSH and TXN could sensitise cancer cells to radiotherapy and chemotherapy both in vitro and in vivo.^12^ At present, most cancer research on IDH2 focuses on its mutation status, such as R172 and R140 mutants in acute myeloid leukaemia^13^ and R172 mutant in glioma.^14^ After searching the cBioPortal database (https://www.cbioportal.org), we found that no IDH mutation existed in 227 ESCC samples. So we focussed on the prognostic and predictive significance of wild-type IDH2 in ESCC. In our previous study, we identified IDH2 as a valuable prognostic marker for ESCC patients who received only surgery.^15^ Although several in vitro studies show that mediating IDH expression could affect the response to γ-rays in human monocytes, glioma cells, and mouse embryonic fibroblasts,^16–18^ animal experiments and studies on the clinical significance remain inadequate. In this study, we probed the effects of IDH2 on radiotherapeutic efficiency and whether IDH2 could be used as a radioresistance indicator in ESCC.

## Methods

### Cell lines and reagents

The human ESCC cell lines Kyse150, Eca109 (China Center For Type Culture Collection, CCTCC), Kyse140, and Kyse510 (donated by Professor Yan Li from Sun Yat-Sen University Cancer Center) were cultured in RPMI 1640 media (Gibco, Life Technologies Inc., Grand Island, NY, USA) supplemented with 10% foetal bovine serum (Gibco). The cell lines were characterised by the Genetic Testing Biotechnology Corporation (Suzhou, China) using short tandem repeat markers and tested for mycoplasma using the Myco-Lumi™ Luminescent Mycoplasma Detection Kit (Beyotime, Nantong, China). Plasmid vectors expressing a short hairpin RNA (shRNA) against IDH2 (sh-IDH2; target sequence: GACCGACTTCGACAAGAATAA) or its counterpart control shRNA (sh-NC; target sequence: GTTCTCCGAAVGTGTCACGT) were transfected into ESCC cells using Lipofectamine 3000 (Invitrogen, Carlsbad, CA, USA), and the stable clones were selected by puromycin. The specificity of sh-IDH2 had been confirmed by rescue experiments in our previous study,^15^ and the efficiency of gene knockdown was measured by quantitative reverse transcriptase–polymerase chain reaction (qRT-PCR). Cells were collected and tested at 48 h after receiving different doses of radiation for various experiments except for the clonal-efficiency assay. TEMPO, cisplatin, and SC-79 were obtained from MCE (Monmouth Junction, USA).

### Quantitative RT-PCR

qRT-PCR was performed as previously described.^15^ Primers were synthesised by Sangon Biotech (Shanghai, China), and the sequences were as follows: IDH2, CAAAAACATCCCACGCCTAGTC and CCCGGTCTGCCACAAAGT; glyceraldehyde 3-phosphate dehydrogenase (GAPDH), GAAGGTCGGAGTCAACGGAT and TGAAACACCGTCTGGCCC.

### Clonal efficiency assay

Clonogenic cell survival was used to calculate the sensitisation enhancement ratio (SER) caused by IDH2 knockdown. The assay was performed as previously described.^15^ The survival curves were obtained with Prism 7.0 (GraphPad Inc., La Jolla, CA, USA). The mean lethal dose (D0), quasi-threshold dose (Dq), and survival fractions at 2 Gy (SF2) were calculated by fitting the survival curves into the single-hit multitarget model (*y* = 1 − [1 − e^(−*kx*)^]^*N*^). The SER of D0 (SER_D0_) and Dq (SER_Dq_) were used to evaluate radioresistance.

### Enzyme assay

The enzymatic activity of IDH2 was assessed by measuring the NADPH/NADP+ ratio in mitochondria.^16^ Mitochondria were extracted using the Cell Mitochondria Isolation Kit (Beyotime), and the NADPH/NADP+ ratio was calculated with the NADP/NADPH-Glo™ Assays (Promega, Madison, WI, USA) according to the manufacturer’s instructions.

### Analysis of cell viability

Cell viability was detected using the cell counting kit-8 (BestBio, Shanghai, China) according to the manufacturer’s instructions. Optical density (OD) values were measured at 450 nm. Cell viability was calculated using the following formula: (OD_experiment_ − OD_blank_)/(OD_control_ − OD_blank_) × 100%.

### Protein extraction and western blot analysis

For in vitro experiments, total protein isolation was performed using the RIPA lysis buffer according to the standard techniques. The mitochondria were removed, and the cytoplasmic protein was extracted using the Cell Mitochondria Isolation Kit (Beyotime) according to the manufacturer’s instruction. For in vivo experiments, the xenograft tissue was flash-frozen in liquid nitrogen and carefully ground, and then the tissue was lysed in RIPA lysis buffer.

Western blot analysis was performed as previously described.^15^ The following primary antibodies were used: anti-IDH2 (1:1000; Proteintech, Chicago, IL, USA), anti-IDH1 antibody (1:1000; Proteintech), anti-GAPDH (1:4000; Proteintech), anti-PRDX1 (1:10000; Abcam, Cambridge, UK), anti-PRDX2 (1:1000; Abcam), anti-PRDX3 (1:10000; Abcam, anti-PRDX4 (1:10,000; Abcam), anti-PRDX5 (1:1000; Abcam), anti-TXN (1:500; Abcam), anti-TXN2 (1:10,000; Abcam), anti-SOD2 (1:1000; Abcam), anti-DRP1 (1:1000; Abcam), anti-OPA1 (1:1000; Abcam), anti-Phospho-AKT (pAKT) (T308) [1:1000; Cell Signaling Technology (CST), Danvers, MA, USA], anti-Bax (1:1000; CST), anti-Bcl-2 (1:1000; CST), anti-cytochrome c (1:1000; CST), anti-cleaved caspase-3 (1:1000; CST), and β-actin (1:2000; Proteintech).

### Cell apoptosis detection

For in vitro experiments, cell apoptosis was detected by flow cytometry using the Phycoerythrin-Annexin V/7-aminoactinomycin D Kit (BD Biosciences, San Jose, CA, USA) according to the manufacturer’s protocol, and the data were analysed using Flowjo 7.6 (Ashland, OR, USA).

To assess cell apoptosis in vivo, terminal deoxynucleotidyl transferase-mediated dUTP-fluorescein nick end labelling (TUNEL) staining was performed using the TUNEL Andy Fluor^TM^ 488 Apoptosis Detection Kit (GeneCopoeia, Inc., Rockville, MD, USA) according to the manufacturer’s instructions. Five fields on each coverslip were randomly pictured using Olympus DP72. The number of TUNEL/4,6-diamidino-2-phenylindole (DAPI)-positive cells was counted using Image-pro plus 6.0 (Media Cybernetics Inc., Silver Spring, MD, USA), and the mean per coverslip was calculated based on three separate experiments.

### ROS measurement

Intracellular ROS and mitochondria-derived superoxide were detected using 2’,7’-dichlorofluorescin diacetate (DCFH-DA) (Sigma-Aldrich) and MitoSOX™ (Invitrogen), respectively. After radiation, Kyse140 and Kyse510 cells were incubated with medium containing 10 μM DCFH-DA for 30 min or 5 μM MitoSOX™ for 10 min at 37 °C under dark conditions. After being rinsed with phosphate-buffered saline (PBS), cells were subjected to flow cytometry (BD Biosciences), and data were analysed using Flowjo 7.6.

### Malondialdehyde (MDA) measurement

Kyse140 and Kyse510 cells were lysed, and the MDA level was measured by the Lipid Peroxidation MDA Assay Kit (Beyotime) according to the manufacturer’s protocol. The MDA content was normalised to milligrams of total protein.

### Immunofluorescence

Cells on coverslips were fixed with paraformaldehyde blocked with goat serum. Then cells were incubated at 4 °C overnight with the primary antibody against 8-hydroxy-2’-deoxyguanosine (8-OHdG) (1:50; Santa Cruz Biotechnology Inc., CA, USA). After rinsing with PBS, coverslips were incubated with fluorescein isothiocyanate-conjugated secondary antibody. Five fields from each coverslip were randomly captured with Olympus DP72, and three independent experiments were performed. Fluorescence density was calculated with Image Pro Plus 6.0.

### Protein carbonyl measurement

After receiving radiation, Kyse140 and Kyse510 cells were lysed, and the protein carbonyl was detected using the Protein Carbonyl Colorimetric Assay Kit (Cayman Chemical, Ann Arbor, USA) according to the manufacturer’s instructions. The carbonyl content was normalised to milligrams of total protein.

### Measurement of mitochondrial membrane potential (∆Ψm)

∆Ψm was measured using the Mitochondrial Membrane Potential Assay Kit with JC-1 (Beyotime) according to the manufacturer’s protocol. The JC-1-loaded cells were subjected to flow cytometry (BD Biosciences), and the shift in fluorescence was analysed with Flowjo 7.6.

### Phosphokinase array

The phosphokinase array was performed using the Human Phospho-Kinase Array Kit (R&D Systems, Minneapolis, USA) according to the manufacturer’s protocol. The relative levels of phosphorylation of 43 kinase phosphorylation sites and 2 related total proteins were detected simultaneously, and the pixel intensity of each spot was measured using ImageJ 1.44 (U.S. National Institutes of Health, Bethesda, MD, USA).

### In vivo experiments

All animal procedures conformed to the guidelines outlined in the Animal Research Reporting In vivo Experiments (ARRIVE; Supplementary Material) and were approved by the ethics committee of Qilu Hospital. The animals were monitored daily for general health status. For the subcutaneous ESCC model, 6-week-old male BALB/c-nu mice were divided into 4 groups: sh-NC, sh-IDH2, sh-NC + R (radiation), and sh-IDH2 + R. Kyse140 cells were resuspended in a 1:1 solution of PBS/Matrigel (BD Biosciences) and injected into the right shoulder of the mice, which were anaesthetised with a xylazine (10 mg/kg) and ketamine (100 mg/kg) mixture by intraperitoneal injection. The sh-NC + R and sh-IDH2 + R groups were given 2 Gy radiation at 3, 5, and 7 days after injection. Tumour volume (*V*) was measured every 3 days in three dimensions (*a*, *b*, *c*) and calculated according to the following formula: *V* = *abc* × 0.52. Mice in the sh-NC and sh-IDH2 groups were sacrificed at 30 days after injection, while the other mice were killed at 45 days after implantation. Mice were killed by cervical dislocation.

### Patients and specimens

A total of 141 patients who underwent dCRT between January 2011 and December 2015 were recruited for this study. The cancer specimens were collected by esophagoscopy before dCRT was performed. Eligible patients had to meet the following criteria: histologically confirmed, no distant metastases except for M1a (supraclavicular or coeliac lymph nodes), and no second primary cancer or previous malignancy. Seventy-four patients were treated at Tengzhou Central People’s Hospital, Shandong Province (T group) while the other 67 patients received treatment at Xuzhou Central Hospital, Jiangsu Province (X group). Regarding chemotherapy, patients in the T group received the docetaxel/cisplatin (TP) regimen, which included docetaxel (25 mg/m^2^) plus cisplatin (25 mg/m^2^) repeated every week for 5 cycles, while those in the X group underwent the 5-fluorouracil/cisplatin regimen, which included cisplatin (75 mg/m^2^) on day 1 plus 5-fluorouracil (1000 mg/m^2^/day) on days 1–4 repeated every 4 weeks for 4 cycles. Concurrent radiotherapy was delivered at a dose of 2 Gy/day, 5 days/week, for a total dose of 60 Gy in 30 fractions. dCRT response (cCR or non-cCR) was evaluated at 4 weeks after the completion of dCRT based on physical examination, oesophagography, computed tomography, esophagoscopy, and biopsy when necessary. cCR, defined as the disappearance of all assessable lesions, was deemed as the criterion of treatment efficacy. Patients who were judged as non-cCR were offered adjuvant chemotherapy or salvage surgery if their condition allowed. Patients were routinely followed up every 3 months in the first 2 years and every 6 months thereafter. The study protocol was approved by the ethics committees of these two institutes. Patients all granted written informed consent for the use of their clinical records.

### Immunohistochemistry (IHC)

IHC was performed as previously described.^15^ The following antibody was used: anti-IDH2 (1:200; Proteintech). Five fields on each slide were randomly imaged, and the scoring was conducted according to the intensity of the dye colour and the number of positive cells by two “blinded” pathologists. The intensity was graded as 0 (no staining), 1 (weak), 2 (moderate), and 3 (intense). The number of positive cells was classified as 0 (<5%), 1 (5–25%), 2 (25–50%), 3 (51–75%), and 4 (>75%). The final score was the multiplication value of these two scores. The level of expression was defined as “high” if the multiplied score was > 8; otherwise it was defined as “low”.

### Statistical analysis

For cell and xenograft experiments, data were presented as the mean ± SD and analysed by two-tailed unpaired *t* test using Prism 7.0.

The interaction between radiation and IDH2 knockdown was analysed using the Bliss independence model.^19^ Based on the inhibition rate (IR), the combined percentage inhibition, IR_R+sh-IDH2_, was predicted using

IR_R+sh-IDH2_ = IR_R_ + IR_sh-IDH2_ − IR_R_ × IR_sh-IDH2_

where IR_R_ and IR_sh-IDH2_ were the IR of mono-treatment with radiation and sh-IDH2, respectively. Then the actual combined percentage inhibition, IR_Actual_, was compared with IR_R+sh-IDH2_. IR_Actual_>, =, and <IR_R+sh-IDH2_ indicated synergistic, additive, and antagonistic effects, respectively.

The relationship between IDH2 expression and the clinicopathological factors was determined by Chi-square test. Overall survival (OS) and progression-free survival (PFS; the time from treatment to disease progression or death from any cause) were assessed by the Kaplan–Meier method and compared with log-rank test. Univariable Cox analysis was applied to evaluate the prognostic significance of parameters. Multivariable analysis by the Cox proportional-hazard model was used to determine the independence of prognostic factors and estimate hazard ratios (HRs) and 95% confidence intervals (CIs). The predictive value of IDH2 expression in the specimen was determined using the receiver-operating characteristic (ROC) curve analysis and the area under the ROC curve (AUC) estimation. Statistical analyses were performed using SPSS (IBM, Armonk, NY, USA), and *P* < 0.05 was considered statistically significant and the inclusion criterion to use a variable from univariable analysis in the multivariable analysis.

## Results

### IDH2 knockdown inhibited radioresistance in ESCC cells

**Fig. 1:**
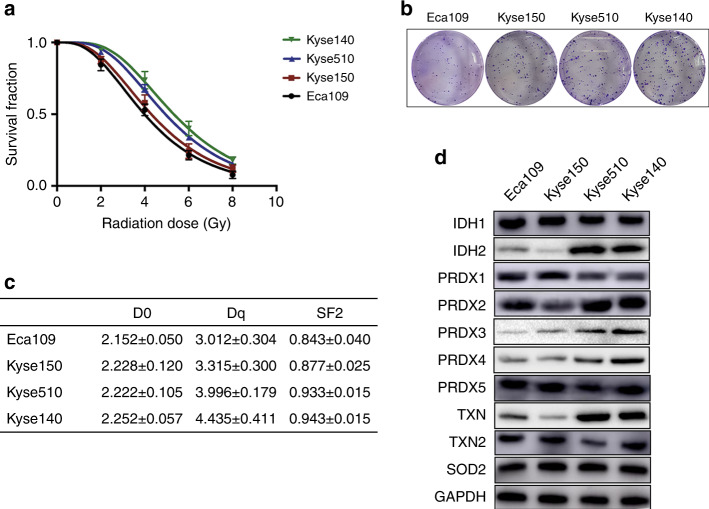
Kyse510 and Kyse140 cells were more radioresistant than Eca109 and Kyse150 cells. Four ESCC cell lines (Eca109, Kyse150, Kyse510, and Kyse140) received different doses of radiation and then the clonal-efficiency assay was performed. **a** Survival curves based on the clonal-efficiency assay. The data shown are the mean ± SD of independent experiments (*n* = 3). **b** Colony formation of ESCC cells exposed to 6 Gy radiation. **c** D0, Dq, and SF2 were calculated using the single-hit multitarget model. **d** Immunoblots of IDH1, IDH2, PRDX1, PRDX2, PRDX3, PRDX4, TXN, TXN2, and SOD2.

Clonal efficiency assay demonstrated that, after radiation, Kyse510 and Kyse140 cells had higher colony formation efficiency than the other two ESCC cell lines. Survival curves were plotted based on the clonal-efficiency assay (Fig. 1a, b), and D0, Dq, and SF2 were calculated. As shown in Fig. 1c, Kyse510 and Kyse140 cells had higher values of Dq and SF2 (all *P* < 0.05) than Eca109 and Kyse150 cells. The above data indicated that Kyse510 and Kyse140 cells were more radioresistant than Eca109 and Kyse150 cells.

Since the antioxidant system plays an important role in radioresistance, we measured the expression of several antioxidant proteins in these four ESCC cells lines. Western blotting showed that the protein levels of PRDX2, PRDX3, PRDX4, and TXN in Kyse510 and Kyse140 cells were higher than those in Eca109 and Kyse150 cells. In addition, Kyse510 and Kyse140 cells also had higher expression and enzymatic activity of IDH2 (Fig. 1d and Supplementary Fig. S1). These data indicate that IDH2-mediated NADPH generation might contribute to radioresistance in Kyse510 and Kyse140 cells.

**Fig. 2:**
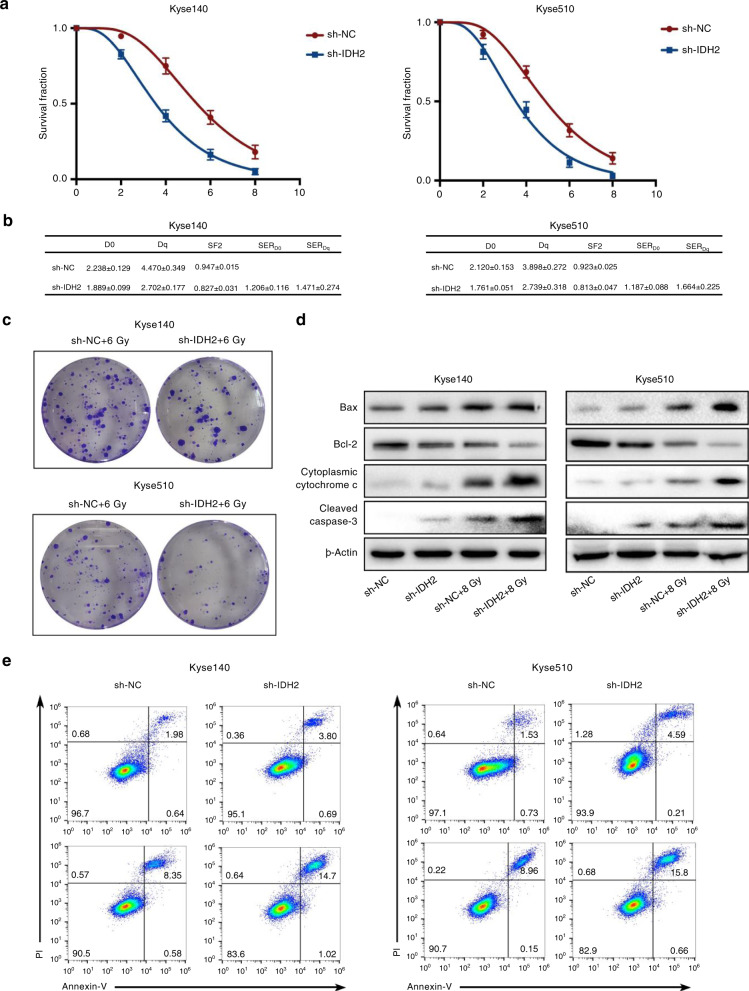
IDH2 knockdown inhibited radioresistance in ESCC cells. Kyse140 and Kyse510 cells were transfected with sh-IDH2 or sh-NC before exposure to different doses of radiation. **a** Survival curves were obtained based on the clonal-efficiency assay. The data shown are the mean ± SD of independent experiments (*n* = 3). **b** D0, Dq, SF2, SER_D0_, and SER_Dq_ were calculated using the single-hit multitarget model. **c** Colony formation of transfected Kyse140 and Kyse510 cells exposed to 6 Gy radiation. **d** Immunoblots of Bax, Bcl-2, cytoplasmic cytochrome c, and cleaved caspase-3. **e** Flow cytometric detection of apoptotic cells.

To further explore the role of IDH2 in radioresistance in vitro, Kyse140 and Kyse510 cells were transfected with sh-IDH2 or sh-NC before exposure to radiation. sh-IDH2 significantly decreased the levels of IDH2 mRNA in Kyse510 and Kyse140 cells (Supplementary Fig. S2). Survival curves showed that, after radiation, cells transfected with sh-NC had higher colony-formation ability than those transfected with sh-IDH2 (Fig. 2a). The characteristics of the survival curves are presented in Fig. 2b. In Kyse140 cells, the sh-NC group had higher D0 (2.238 ± 0.129 vs. 1.889 ± 0.099), Dq (4.470 ± 0.349 vs. 2.702 ± 0.177), and SF2 (0.947 ± 0.015 vs. 0.827 ± 0.031) values than the sh-IDH2 group (All *P* < 0.05). The SER_D0_ and SER_Dq_ for IDH2 knockdown in Kyse140 cells were 1.206 ± 0.116 and 1.471 ± 0.274, respectively. The same results were also observed in Kyse510 cells and the “relatively radiosensitive” cells (Eca109 and Kyse150 cells) (Supplementary Fig. S3). Then the expression levels of the key proteins in mitochondria-mediated apoptosis were analysed. Radiation exposure increased the Bax expression and decreased the Bcl-2 level, thus increasing the Bax/Bcl-2 ratio. In addition, the protein levels of cleaved caspase-3 and cytoplasmic cytochrome c were also increased after radiation. IDH2 knockdown intensified the radiation-induced increase in Bax/Bcl-2 ratio, cytochrome c release, and caspase-3 cleavage (Fig. 2d). Cell apoptosis was measured by flow cytometry (Fig. 2e). After radiation, the apoptosis rate of Kyse140 cells was increased from 2.16 ± 0.57% to 8.71 ± 0.64% (*P* < 0.05). IDH2 knockdown exacerbated the radiation-induced apoptosis, raising the apoptosis rate to 15.08 ± 0.72% (*P* < 0.05). The same results were also observed in Kyse510 cells (all *P* < 0.05) (Supplementary Fig. S4A). These results indicate that IDH2 plays an important role in radioresistance in ESCC cells.

### IDH2 knockdown further aggravated radiation-induced oxidative stress in ESCC cells

**Fig. 3:**
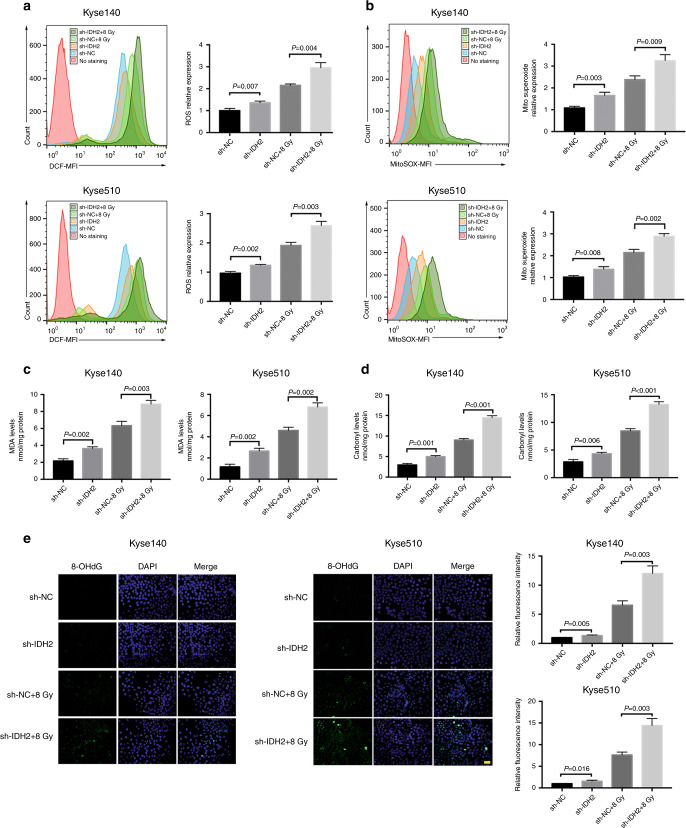
IDH2 knockdown further aggravated the radiation-induced oxidative stress in ESCC cells. Transfected Kyse140 and Kyse510 cells were subjected to 0 or 8 Gy radiation at 48 h before detection. Flow cytometric detection of **a** intracellular ROS and **b** mitochondria-derived superoxide. Contents of **c** MDA and **d** protein carbonyl in Kyse140 and Kyse510 cells. **e** Detection of 8-OHdG by immunofluorescence staining and quantification analysis of immunofluorescence. Nuclei were counterstained with DAPI. Scale bar = 20 μm. The data shown are the mean ± SD of independent experiments (*n* = 3). *P* value was calculated by two-tailed unpaired *t* test.

**Fig. 4:**
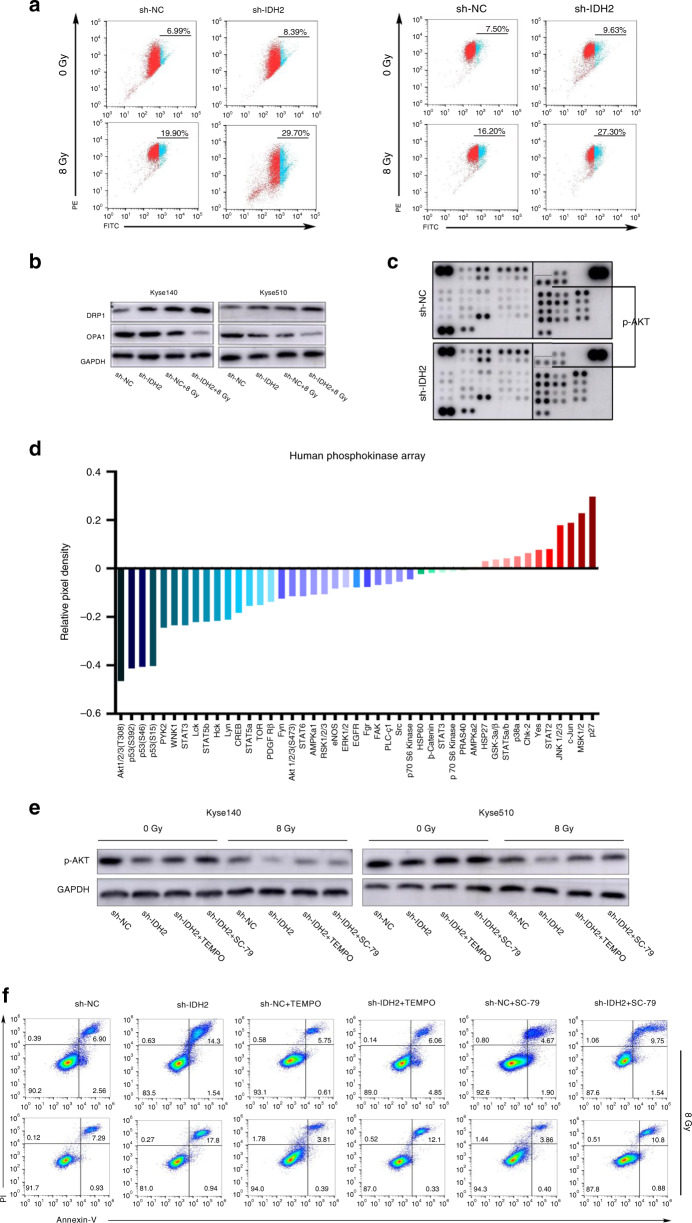
IDH2 knockdown exacerbated the radiation-induced mitochondrial dysfunction and inhibition of AKT phosphorylation contributed to the IDH2 knockdown-induced radiosensitisation. **a** Flow cytometric detection of ∆Ψm. **b** Immunoblots of DRP1 and OPA1. **c** Phosphokinase array of 43 phosphorylated kinases in Kyse140 cells transfected with sh-IDH2 or sh-NC. **d** Quantification of the phosphokinase array. **e** Immunoblots of p-AKT. **f** Flow cytometric detection of apoptotic cells.

In Kyse140 cells, IDH2 knockdown significantly enhanced the content of intracellular ROS and mitochondrial superoxide in both the steady state and after ROS induction by radiation. The same effects were also observed in Kyse510 cells (All *P* < 0.05, Fig. 3a, b). In addition, the sh-IDH2-mediated radiosensitivity was compromised by TEMPO, a selective scavenger of mitochondrial ROS (Fig. 4f). These results indicate that IDH2 knockdown promotes radiosensitivity via inducing an imbalance in the intracellular redox state. Moreover, we found that cisplatin induced oxidative stress, and sh-IDH2 also promoted its therapeutic effects in ESCC cells (Supplementary Fig. S5).

### IDH2 knockdown intensified radiation-induced oxidative damage in ESCC cells

In Kyse140 cells, radiation significantly increased the levels of MDA and protein carbonyl, which were further amplified by IDH2 knockdown (Fig. 3c, d). 8-OHdG was detected by immunofluorescence, as shown in Fig. 3e. Kyse140 cells in the sh-NC group exhibited weak green fluorescence, while radiation enhanced the fluorescent density. IDH2 knockdown caused higher fluorescent density in the sh-IDH2 + 8 Gy group than that in the sh-NC + 8 Gy group. The same effects were also observed in Kyse510 cells. These results show that IDH2 knockdown could aggravate the oxidative injury caused by radiation.

### IDH2 knockdown exacerbated radiation-induced mitochondrial dysfunction

Mitochondrial function was evaluated by measuring ∆Ψm. As shown in Fig. 4a and Supplementary Fig. S4B, radiation exposure significantly decreased ∆Ψm in Kyse140 cells and IDH2 knockdown further amplified that effect. In addition, radiation increased the expression of dynamin-related protein 1 (DRP1), the central mediator of mitochondrial fission, whereas it decreased the protein level of optic atrophy 1 (OPA1), which is required for inner membrane fusion. IDH2 knockdown amplified the radiation-induced increase in DRP1 expression and decrease in OPA1 protein level (Fig. 4b). The same effects of IDH2 knockdown were also observed in Kyse510 cells.

### Inhibition of AKT phosphorylation mediated IDH2 knockdown-induced radiosensitivity

To illuminate the mechanisms underlying IDH2 knockdown-mediated radiosensitisation, we measured the levels of 43 phosphorylated kinases in lysates from cells transfected with sh-IDH2 or sh-NC using a phosphokinase array. In response to IDH2 knockdown, we identified inhibition in the phosphorylation status of several kinases, including pAKT (T308), phospho-P53 (pP53) (S392), pP53 (S46), and pP53 (S15) (Fig. 4c). As shown in Fig. 4d, the decrease in pAKT (T308) phosphorylation was the most significant. The attenuation in AKT activation after IDH2 knockdown was further confirmed by western blotting. Radiation inhibited AKT phosphorylation, and the antioxidant TEMPO could alleviate the IDH2 knockdown-induced decrease in AKT phosphorylation in both the steady state and after ROS induction by radiation (Fig. 4e). These results indicated that IDH2 knockdown inhibited AKT phosphorylation in a ROS-dependent manner. Our previous study had proved the vital role of AKT activation in radioresistance of ESCC cells.^20^ So we examined whether the IDH2 knockdown-induced radiosensitivity was dependent on the attenuation of AKT phosphorylation. As shown in Fig. 4f, the selective AKT agonist SC-79^21^ inhibited the sh-IDH2-induced radiosensitisation in Kyse140 cells (Supplementary Fig. S4C). The same results were also observed in Kyse510 cells.

### IDH2 knockdown enhanced the efficacy of radiotherapy in vivo

**Fig. 5:**
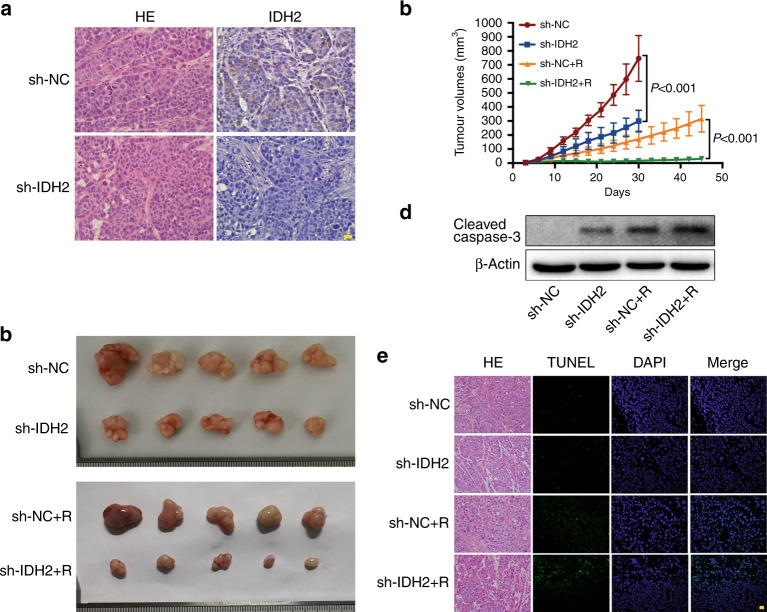
IDH2 knockdown induced radiosensitisation in murine xenografts. **a** HE staining and IHC images of IDH2 of murine xenografts. Nuclei were counterstained with haematoxylin. Scale bar = 20 μm. **b** Tumours in the sh-NC and sh-IDH2 groups were harvested on day 30, while tumours in the sh-NC + R and sh-IDH2 + R groups were harvested on day 45. **c** Tumour volume was recorded every 3 days and presented as mean ± SD (*n* = 5). *P* value was calculated by two-tailed unpaired *t* test. **d** Immunoblots of cleaved caspase-3. **e** HE and TUNEL staining of murine xenografts. Nuclei were counterstained with haematoxylin and DAPI, respectively. Scale bar = 20 μm.

To further test the sensitisation effect of IDH2 knockdown on radiotherapy in vivo, nude mice were subcutaneously implanted with Kyse140 cells transfected with sh-IDH2 or sh-NC. All mice (5/5 for each group) were included in the analysis without any significant adverse event. The immunostaining of IDH2 in xenografts is shown in Fig. 5a. In tumours derived from sh-IDH2-transfected cells, the IDH2 expression was significantly decreased. As shown in Fig. 5b, c, mice in the sh-IDH2 + R group had lower tumour volumes than those in the sh-NC + R group. Western blot analysis showed that radiation enhanced the protein level of cleaved caspase-3 and that effect was intensified by IDH2 knockdown (Fig. 5d). TUNEL staining revealed that there were more TUNEL-positive cells in the sh-IDH2 + R group than that in the sh-NC + R group (Fig. 5e). In addition, the Bliss independence model showed that radiation and IDH2 knockdown induced synergistic inhibition in cell viability and tumour growth (Supplementary Fig. S6).

### Correlation between IDH2 expression and dCRT response

**Fig. 6:**
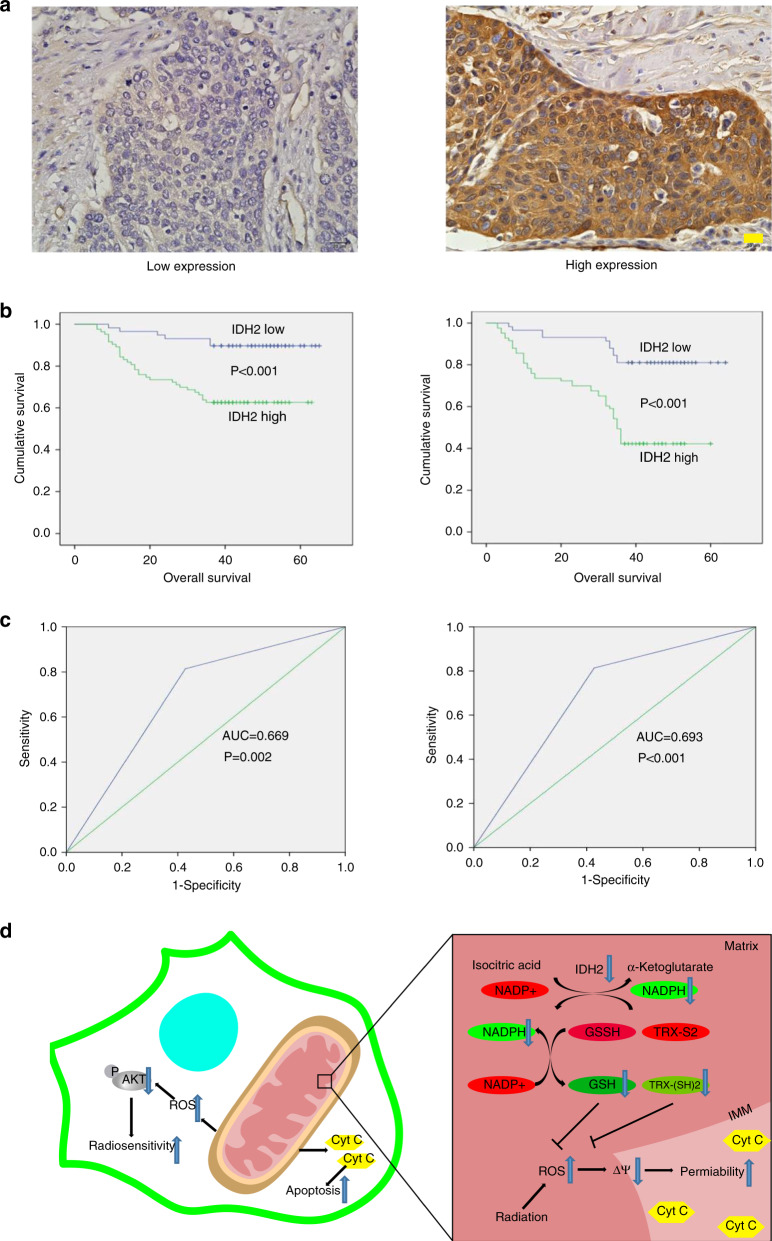
Kaplan–Meier curve and receiver operating curve (ROC) analyses of IDH2. **a** IHC images of IDH2 in biopsy specimens from ESCC patients. Nuclei were counterstained with haematoxylin. Scale bar = 20 μm. **b** Kaplan–Meier curve for overall survival (OS) and progression-free survival (PFS) of patients with high IDH2 expression (*n* = 83) and low IDH2 expression (*n* = 58). *P* value was calculated by log-rank test. **c** ROC curve analysis for OS and PFS. Area under the ROC curve (AUC) estimation was applied to assess the predictive ability of IDH2. **d** The mechanism of the radiosensitisation effect of IDH2 knockdown in ESCC cells.

The IDH2 expression in biopsy specimens was detected by IHC. As shown in Fig. 6a, IDH2 was mainly located in the cytoplasm. Among the 141 ESCC patients, 58 expressed low levels of IDH2 expression and 83 expressed high levels of IDH2. The correlation between clinicopathological variables and IDH expression is presented in Supplementary Table S1. At the evaluation time, cCR and non-cCR were achieved in 41 and 100 patients, respectively. IDH2 expression was significantly correlated with dCRT response (*P* = 0.009), in which low IDH2 expression was observed more frequently in the cCR group than in the non-cCR group (59% vs. 34%).

### Correlation between IDH2 expression and ESCC patient survival

Kaplan–Meier curve showed that lower IDH2 expression was related to better OS and PFS (Fig. 6b). In univariate analysis, IDH2 upregulation correlated with decreased OS (*P* = 0.001) and PFS (*P* < 0.001). Multivariate analyses further confirmed that IDH2 was an independent prognostic indicator for OS (*P* = 0.004) and PFS (*P* = 0.001). In addition, the T and N stages were also predictive factors for OS and PFS (Supplementary Table S2). ROC analyses were performed for the Cox regression model. The AUC value of IDH2 for OS was 0.669 (*P* = 0.002) and for PFS was 0.693 (*P* < 0.001) (Fig. 6c).

## Discussion

Currently, when oncologists make treatment plans for ESCC patients, baseline clinical staging is still the major concern, while the tumour heterogeneity has not been taken into consideration. However, therapeutic efficacy varies largely among patients, even when they are diagnosed at the same stage. Therefore, personalised approaches to the diagnosis and treatment of ESCC patients are urgently needed. In this study, we focussed on enhanced biomarker detection to predict radiotherapy efficacy and identify appropriate patients who were most likely to benefit from radiation.

Since ESCC is a complex and heterogeneous disease, selecting the right ESCC cell line as the experimental model is critical for defining the importance of IDH2 in radioresistance. Our data showed that, after radiation, Kyse510 and Kyse140 cells had higher colony formation efficiency than Eca109 and Kyse150 cells, indicating that these cells were more radioresistant. ROS is the initial mediator of radiation-induced cell death, and the antioxidant system is intimately associated with radioresistance. To further explore the differences in the activity of the antioxidant system, we performed western blot analysis on several antioxidant proteins in these cell lines. As might be expected, Kyse510 and Kyse140 cells had significantly increased levels of PRDX2, PRDX3, PRDX4, and TXN in comparison to Eca109 and Kyse150 cells. In addition, expression and enzymatic activity of IDH2 were also increased in Kyse510 and Kyse140 cells. These results indicated that the IDH2-maintained antioxidant system might play a vital role in radioresistance of ESCC cells.

The role of IDH2 in radioresistance was further probed by IDH2 knockdown in vitro and in vivo. Flow cytometry and clonal-efficiency assay showed that IDH2 knockdown further promoted the radiation-induced apoptosis and inhibition in colony formation, indicating the facilitation of IDH2 in radioresistance. This effect was also validated by animal experiments. Compelling evidence has indicated that mitochondria-mediated cell apoptosis is tightly controlled by the Bcl-2 family, which is composed of pro-apoptotic proteins, including Bax and Bak, and anti-apoptotic proteins, such as Bcl-2 and Bcl-xl.^22^ During apoptosis, Bax is inserted into the outer mitochondrial membrane (OMM) and increases its permeability, leading to a leakage of cytochrome c into the cytoplasm, where it activates caspase-3, the implementer of apoptosis. The anti-apoptotic BCL-2 proteins could inhibit the permeabilisation of OMM, thus restraining the apoptotic effects.^23^ Previous studies have indicated that the increased ratio of Bax to Bcl-2 is a major driving force in mitochondrial dysfunction and subsequent apoptosis in anticancer treatments.^24^ Our results showed that IDH2 knockdown promoted the radiation-induced increase in the Bax/Bcl-2 ratio, cytochrome c release, and caspase-3 cleavage, indicating that mitochondria-meditated apoptosis contributed to the IDH2 knockdown-induced radiosensitisation.

Radiation damage is primarily mediated by ROS that are generated after water radiolysis.^25^ We found that radiation exposure induced the overload of intracellular ROS and mitochondrial superoxide, leading to the oxidation of lipids, protein, and DNA, as evidenced by the increase in the contents of MDA, protein carbonyl, and 8-OHdG. Moreover, IDH2 knockdown intensified the radiation-induced oxidative damage. Exogenous antioxidant (TEMPO) attenuated the IDH2 knockdown-induced radiosensitisation, indicating the vital importance of IDH2 in the antioxidant defence system against radiation-induced oxidative stress in ESCC cells. In addition, we found that cisplatin induced oxidative stress, and IDH2 knockdown promoted its therapeutic effects in ESCC cells. Given that cisplatin is a commonly used chemotherapeutic agent for ESCC, targeting IDH2 may bring great benefits to patients who receive cisplatin–radiation combination treatment. These data indicate that IDH2 knockdown may facilitate the efficacy of any treatment that induces oxidative stress, thus broadening the application prospect of manipulating IDH2 expression.

One of the characteristics associated with oxidative stress is mitochondrial dysfunction,^26^ which has been proved to play a key role in response to radiotherapy.^27,28^ We found that IDH2 knockdown exacerbated the radiation-induced mitochondrial dysfunction, as evidenced by the decrease in ∆Ψm. Mitochondrial dysfunction could also induce ROS overproduction, thereby creating a vicious positive feedback cycle.^29^ In addition, excessive ∆Ψm reduction represents the hyperpermeability of OMM, which contributes to cytochrome c release, causing mitochondria-dependent apoptosis.^30^ Mitochondria exist as a dynamic network that often changes size and subcellular distribution. Their dynamics is a result of coordinated fission and fusion, which are mainly regulated by DRP1 and OPA1, respectively.^31,32^ Compelling evidence has shown that cells with a highly connected mitochondrial network maintain higher ∆Ψm and generate more ATP than cells with fragmented mitochondria. In addition, cells with hyperfused mitochondria are less vulnerable to cellular stresses.^33^ Our data showed that, after radiation, cells transfected with sh-IDH2 had higher expression of DRP1 and a lower protein level of OPA1, indicating that there were more impaired mitochondria in these cells. The above results showed that mitochondrial dysfunction contributed to IDH2 knockdown-induced radiosensitisation.

AKT, as an important survival kinase, has been proved to play a key role in radioresistance in several neoplasms.^34–36^ Our previous study demonstrated that AKT phosphorylation contributed to radioresistance of ESCC cells.^20^ In addition, AKT phosphorylation is responsive to oxidative stress.^37^ In this study, inhibition of AKT phosphorylation was identified as an underlying molecular occurrence after mediating IDH2 activity. Exogenous antioxidant restored that molecular occurrence and AKT activator alleviated IDH2 knockdown-induced radiosensitisation. These findings indicate that IDH2 knockdown enhances radiosensitivity through ROS-dependent inhibition of the AKT signalling pathway. In addition to AKT dephosphorylation, inhibition of P53 phosphorylation was also observed. The underlying mechanism is not clear. Several kinases have been implicated in P53 phosphorylation, including ataxia-telangiectasia mutated protein, extracellular signal-regulated kinases, and p38 mitogen-activated protein kinase.^38^ Considering that those kinases are common downstream effectors of pathways that respond to genotoxic stress, we hypothesise that IDH2 knockdown-induced DNA damage may affect the activity of the regulatory kinase, thus inhibiting P53 phosphorylation. The exact mechanism and potential effects of P53 dephosphorylation awaits further studies.

In clinical study, IDH2 expression showed significant correlation with OS and PFS in ESCC patients. Although IDH2 expression was found to be associated with dCRT response and T stage, the predictive value remained significant after adjustment for other clinical characteristics in multivariable analysis. According to the ROC analysis, IDH2 expression could accurately predict OS and PFS in ESCC. These results raise the possibility that IDH2 could be an indicator for radioresistance in ESCC and targeting IDH2 might represent a promising strategy for radiosensitisation in ESCC.

## Supplementary information

Supplementary Materials

## Data Availability

The data presented in the current study are available upon reasonable request from the corresponding author.
