# Supplementary material for: Isocitrate dehydrogenase 2 contributes to radiation resistance of oesophageal squamous cell carcinoma via regulating mitochondrial function and ROS/pAKT signalling
Source: Br J Cancer. 2020 May 5;123(1):126–36. doi: 10.1038/s41416-020-0852-4 (PMC7340793; doi:10.1038/s41416-020-0852-4)
Supplement: Supplementary file 1 — Supplementary Materials [file 41416_2020_852_MOESM1_ESM.doc]

**Supplementary Table S1. The correlation of clinicopathological variables of ESCC with IDH2** expression

| Clinicopathological features | IDH2 expression | | *P*^a^ value |
| --- | --- | --- | --- |
|  | Low (n=58) | Relatively high (n=83) |  |
| Age |  |  | 0.150 |
| <65 | 24 | 24 |  |
| ≥65 | 34 | 59 |  |
| Gender |  |  | 0.725 |
| Male | 36 | 54 |  |
| Female | 22 | 29 |  |
| Pre-treatment Clinical T stage |  |  | 0.016 |
| 2 | 18 | 10 |  |
| 3 | 32 | 54 |  |
| 4 | 8 | 19 |  |
| Pre-treatment Clinical N stage |  |  | 0.425 |
| 0 | 13 | 11 |  |
| 1 | 26 | 36 |  |
| 2 | 16 | 29 |  |
| 3 | 3 | 7 |  |
| M1a status |  |  |  |
| No | 50 | 78 | 0.144 |
| Yes | 8 | 5 |  |
| Histological differentiation |  |  | 0.897 |
| Well | 27 | 39 |  |
| Moderate | 22 | 29 |  |
| Poor | 9 | 15 |  |
| Tumour location |  |  |  |
| Upper third | 21 | 26 | 0.598 |
| Middle third | 27 | 37 |  |
| Lower third | 10 | 20 |  |
| dCRT response |  |  | 0.009 |
| Non-cCR | 34 | 66 |  |
| cCR | 24 | 17 |  |

*P*^a^: Chi-square test.

**Supplementary Table S2. Univariate and multivariate analyses of prognostic variables**

|  | OS  Univariate  analysis | | | OS | | | | | PFS  Univariate analysis | | PFS  Multivariate  analysis | | |  | | | | | | | | |
| --- | --- | --- | --- | --- | --- | --- | --- | --- | --- | --- | --- | --- | --- | --- | --- | --- | --- | --- | --- | --- | --- | --- |
|  |  |  |  | Multivariate  Analysis | | | | |  |  |  |  |  |  | | | | | | | | |
| Variable *P* value | | | | *P* value | | HR | 95%CI | | *P* value | | *P* value | HR | 95%CI |  | | | | | | | | |
| Gender (Female VS. Male) | | | 0.208 |  | |  | |  | 0.225 | |  |  |  |  | | | | | | | | |
| Age (<65 vs. ≥65) | | | 0.820 |  |  | | |  | 0.814 | |  |  |  |  | | | | | | | | |
| T stage | | | 0.001 | <0.001 | |  | |  | <0.001 | | <0.001 |  |  |  | | | | | | | | |
| T2 | | |  |  | | 1.000 | | Reference |  | |  | 1.000 | Reference |  | | | | | | | | |
| T3 | | |  | 0.849 | | 0.883 | | 0.244-3.187 |  | | 0.618 | 0.776 | 0.286-2.106 |  | | | | | | | | |
| T4 | | |  | 0.064 | | 3.426 | | 0.932-12.601 |  | | 0.066 | 2.590 | 0.938-7.155 |  | | | | | | | | |
| N stage | | | <0.001 | <0.001 | |  | |  | <0.001 | | <0.001 |  |  |  | | | | | | | | |
| N0 | | |  |  | | 1.000 | | Reference |  | |  | 1.000 | Reference |  | | | | | | | | |
| N1 | | |  | 0.600 | | 0.655 | | 0.134-3.190 |  | | 0.414 | 0.686 | 0.279-1.691 |  | | | | | | | | |
| N2 | | |  | 0.224 | | 2.520 | | 0.568-11.182 |  | | 0.754 | 0.868 | 0.357-2.110 |  | | | | | | | | |
| N3 | | |  | 0.021 | | 6.610 | | 1.328-32.901 |  | | 0.003 | 5.064 | 1.755-14.605 |  | | | | | | | | |
| Differentiation | | | 0.003 | <0.001 | |  | |  | 0.038 | | <0.001 |  |  |  | | | | | | | | |
| Well | | |  |  | | 1.000 | | Reference |  | |  | 1.000 | Reference |  | | | | | | | | |
| Moderate | | |  | 0.106 | | 2.008 | | 0.863-4.673 |  | | 0.792 | 1.090 | 0.573-2.074 |  | | | | | | | | |
| Poor | | |  | <0.001 | | 7.267 | | 2.843-18.577 |  | | 0.001 | 3.661 | 1.694-7.911 |  | | | | | | | | |
| Tumor location | | | 0.911 |  | |  | |  | 0.492 | |  |  |  |  | | | | | | | | |
| Upper third | | |  |  | |  | |  |  | |  |  |  |  | | | | | | | | |
| Middle third | | |  |  | |  | |  |  | |  |  |  |  | | | | | | | | |
| Lower third | | |  |  | |  | |  |  | |  |  |  |  | | | | | | | | |
| Chemotherapy regimens | | | 0.702 |  | |  | |  | 0.458 | |  |  |  |  | | | | | | | | |
| (5-fluorouracilplus cisplatin vs. docetaxel plus cisplatin) | | |  |  | |  | |  |  | |  |  |  |  | | | | | | | | |
| CRT response 0.003 0.014 6.447 1.458-28.514 <0.001 0.001 11.737 2.734-50.381 | | | | | | | | | | | | | |  | |  | 4.705 | 1.320-16.769 | 0.001 | 0.001 | 8.767 | 2.641-29.108 |
| (non-cCR vs. cCR) | |  | |  | |  | |  |  |  | |  |  | |  | | | | | | | |
| IDH2 expression | | 0.001 | | 0.004 | | 3.860 | | 1.547-9.629 | <0.001 | 0.001 | | 3.059 | 1.562-5.991 | |  | | | | | | | |
| (High vs. relatively low) | |  | |  | |  | |  |  |  | |  |  | |  | | | | | | | |

Abbreviations: OS, overall survival; PFS, progression-free survival; CI: confidence interval.


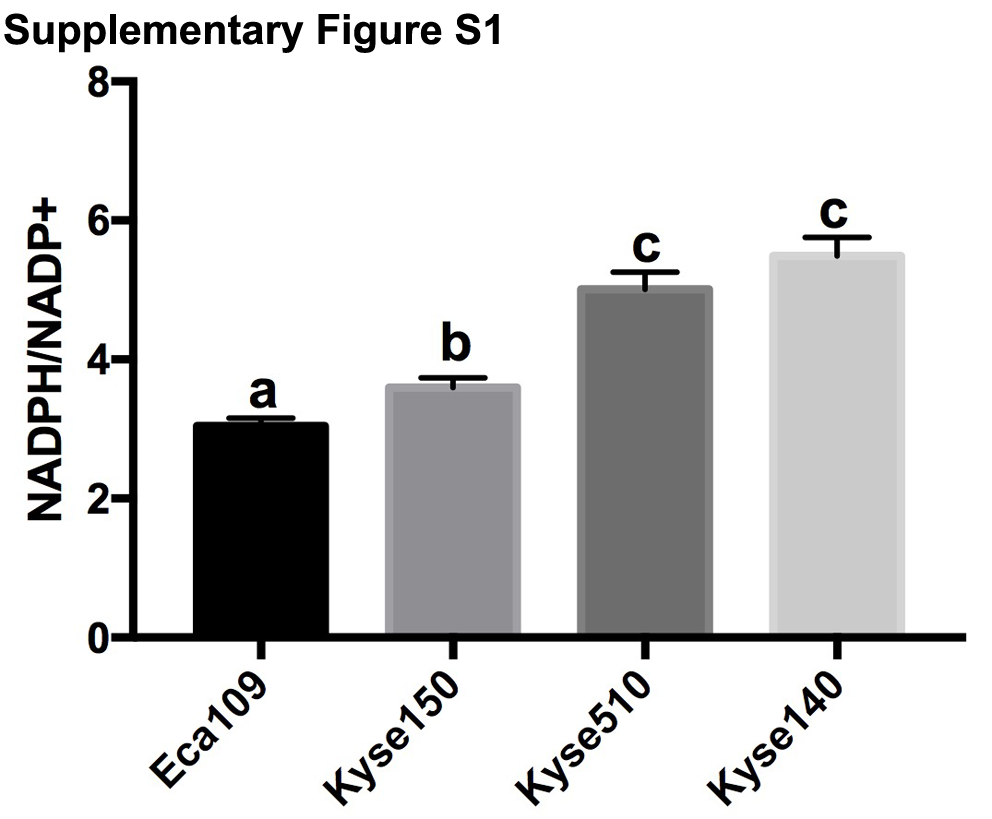


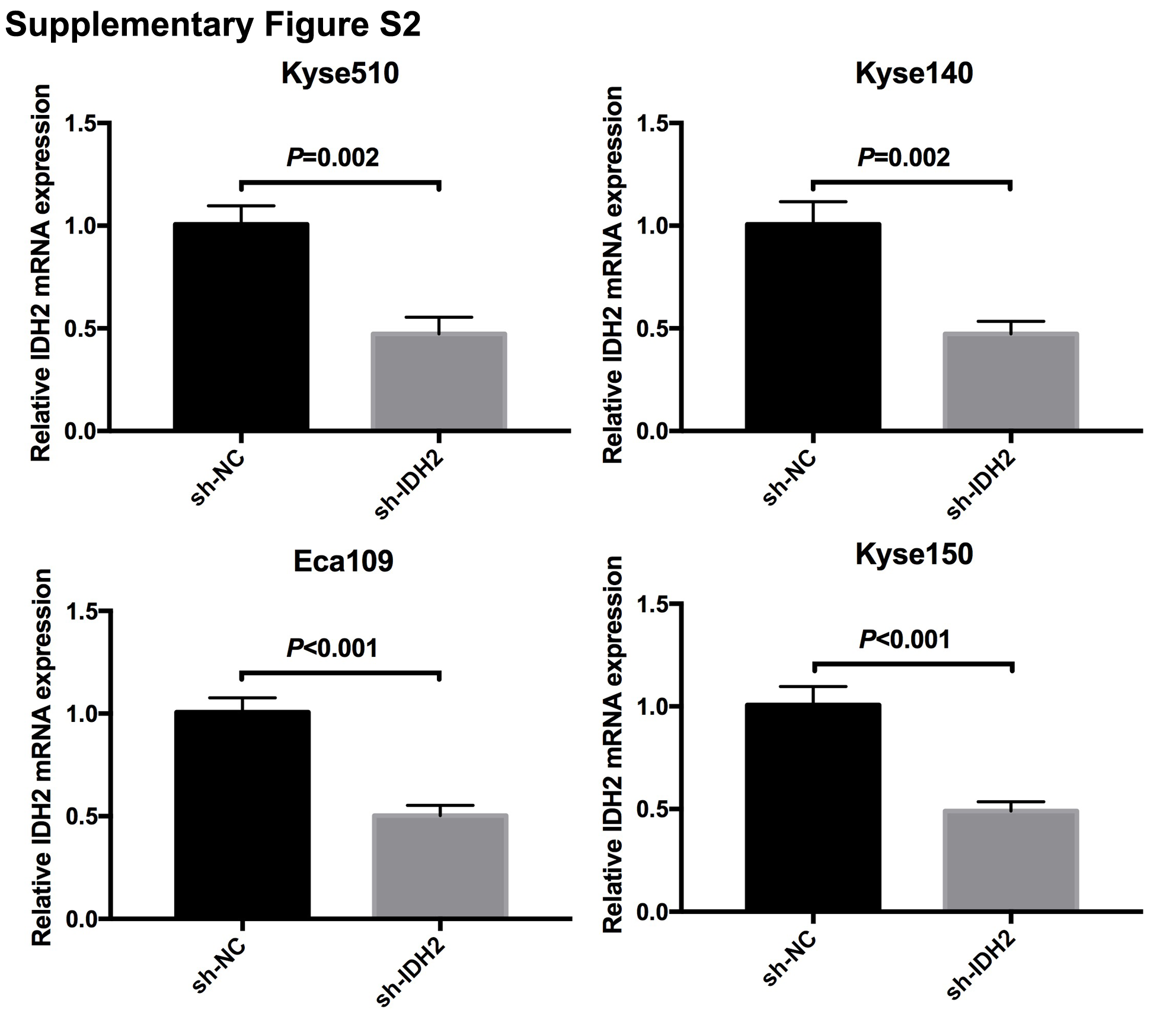


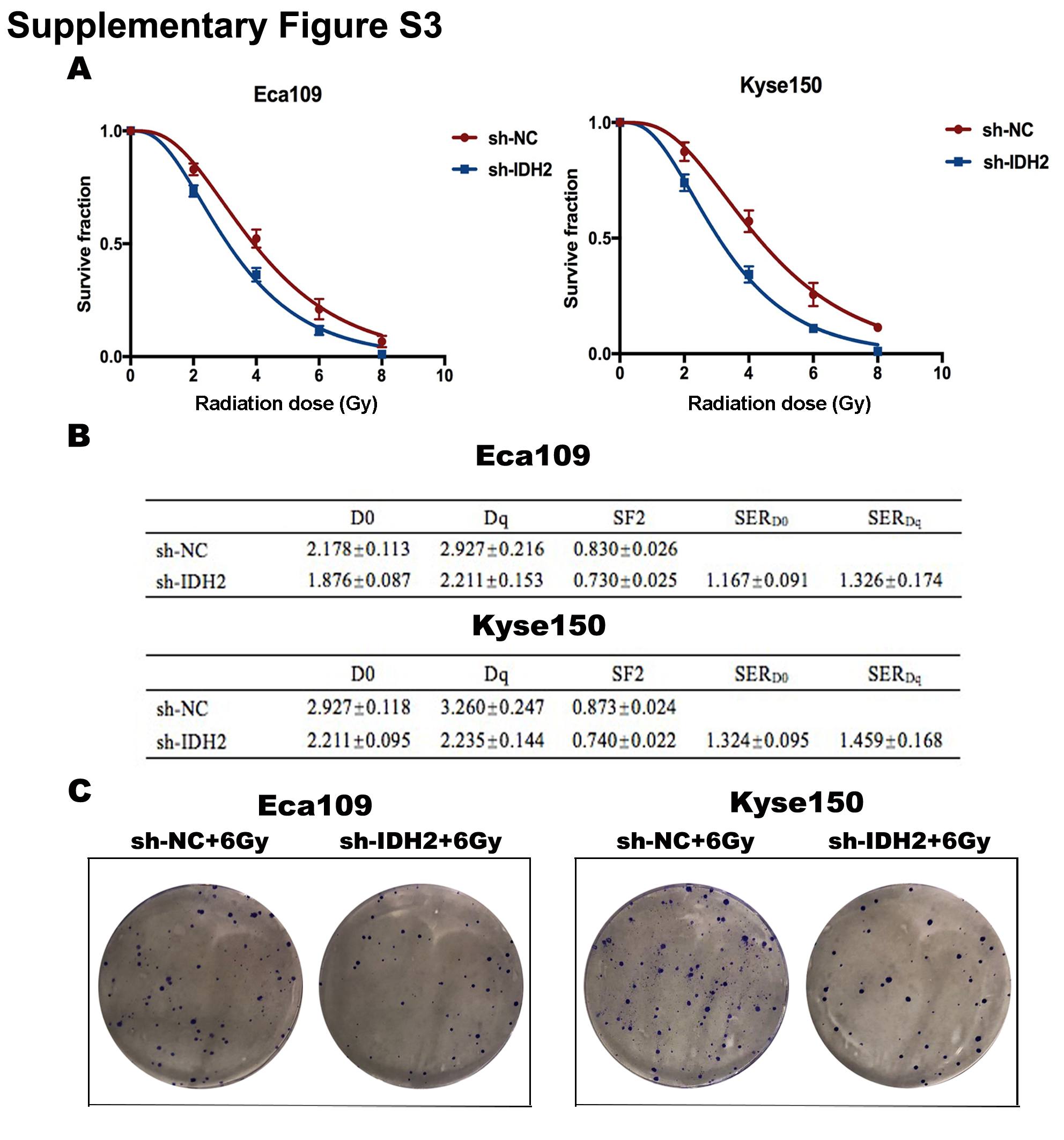


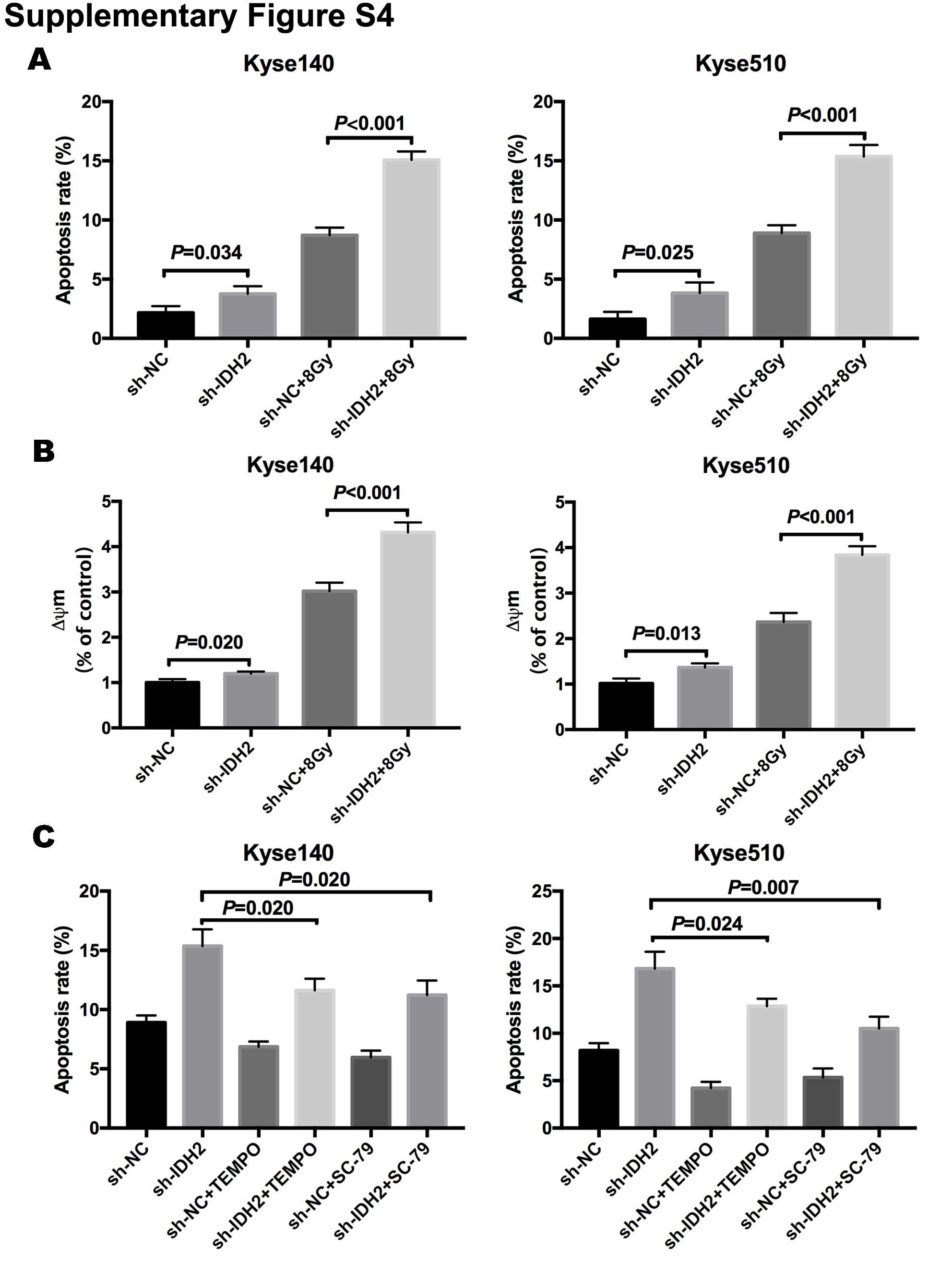


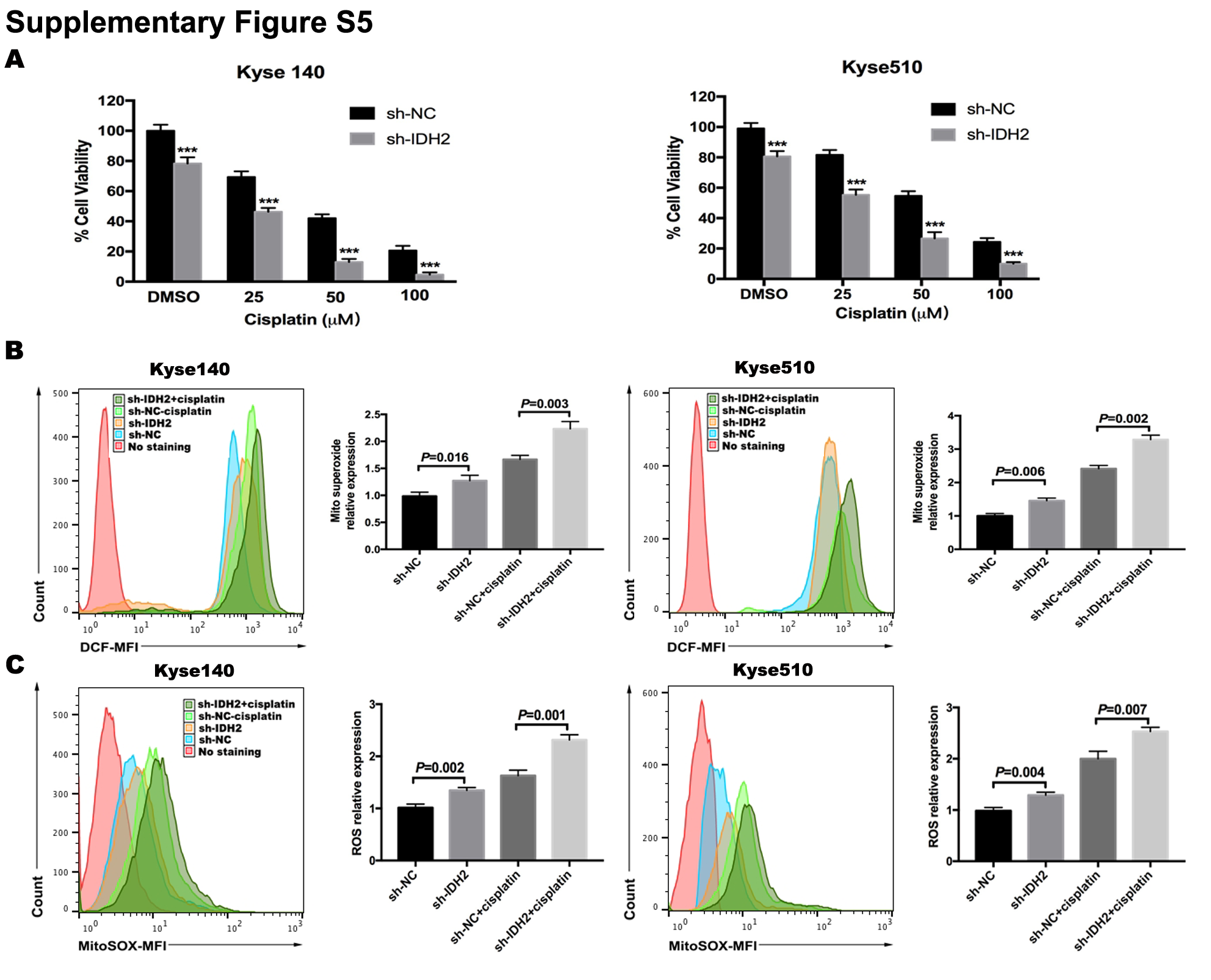


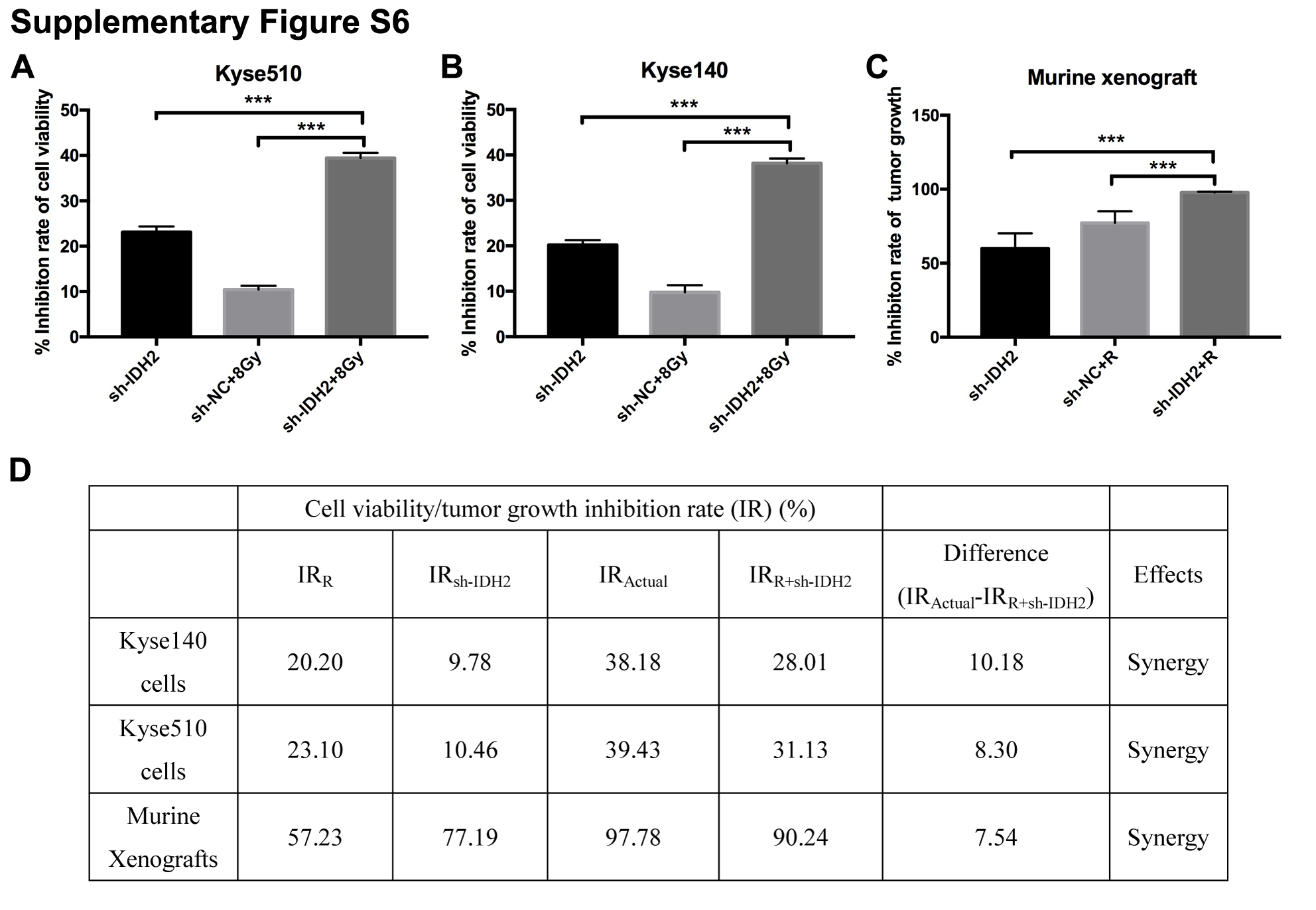


**Supplementary Figure Legends**

**Supplementary Figure S1:** Kyse510 and Kyse140 cells had higher enzymatic activity of IDH2 than Eca109 and Kyse150 cells. The NADPH/NADP+ ratio in Eca109, Kyse150, Kyse510 and Kyse140 cells. The data shown are the mean ± SD of independent experiments (n=3). Statistical significance determined by the two-tailed unpaired t-test and indicated with letters above (P < 0.05). Groups that share the same letter are not significantly different.

**Supplementary Figure S2:** IDH2 was knocked down by sh-IDH2 in ESCC cells. qRT-PCR analysis of IDH2 mRNA expression in Kyse510, Kyse140, Eca109, and Kyse150 cells. The data shown are the mean ± SD of independent experiments (n=3). P value was calculated by two-tailed unpaired t-test.

**Supplementary Figure S3:** The radiosensitization effect of IDH2 knockdown in Eca109 and Kyse150 cells. (A) Survival curves were obtained based on the clonal efficiency assay. The data shown are the mean ± SD of independent experiments (n=3). (B) D0, Dq, SF2, SERD0, and SERDq were calculated using the single-hit multitarget model. (C) Colony formation of transfected Eca109 and Kyse150 cells exposed to 6 Gy radiation.

**Supplementary Figure S4:** Statistic analysis on flow cytometry. (A) Statistical analysis of Fig. 2E. (B) Statistical analysis of Fig. 4A. (C) Statistical analysis of Fig. 4F. The data shown are the mean ± SD of independent experiments (n=3). P value was calculated by the two-tailed unpaired t-test.

**Supplementary Figure S5:** IDH2 knockdown also promoted the therapeutic effects of cisplatin in ESCC cells. (A) Kyse140 and Kyse510 cells transfected with sh-NC or sh-IDH2 were treated with different doses of cisplatin, and then cell viability was detected by CCK-8 assay. The data shown are the mean ± SD of independent experiments (n=3). *** P<0.001, two-tailed upaired t-test. Flow cytometric detection of (B) intracellular ROS and (C) mitochondria-derived superoxide. The data shown are the mean ± SD of independent experiments (n=3). P value was calculated by the two-tailed unpaired t-test.

**Supplementary Figure S6:** IDH2 knockdown and radiation induced synergistic inhibition in cell viability and murine xenograft growth. (A, B) Kyse510 and Kyse140 cells transfected with sh-NC or sh-IDH2 were exposed to 8Gy radiation, and then cell viability was detected by CCK-8 assay. The inhibition rate (IR) on cell viability was calculated and presented as mean ± SD (n=3). *** P<0.001, two-tailed upaired t-test. (C) The IR on xenograft growth was calculated based on the tumor volume and shown as mean ± SD (n=5). *** P<0.001, two-tailed upaired t-test. (D) Effects of radiation and IDH2 knockdown combination on cell viability and xenografts growth evaluated by the Bliss independence model.

**Supplementary Methods**

Animal experiments in compliance with the ARRIVE guidelines

| Ethical statement | All experimental procedures were approved by the Ethics Committee of Qilu Hospital of Shandong University and animal care was closely monitored in conformity with the Guide for the Care and Use of Laboratory Animals of the National Institutes of Health. |
| --- | --- |
| Study design | - Number of experimental groups: for the subcutaneous ESCC model, 20 mice were randomly assigned to 4 groups: sh-NC, sh-IDH2, sh-NC+R (radiation), and sh-IDH2+R (5 mice per group, randomization was assigned by a computer-based, Excel-generated list of all mice). - Experimental unit: 6-week-old male BALB/c-nu mice. |
| Experimental procedures | - Xenograft model establishment: Kyse140 cells were resuspended in a 1:1 solution of PBS/Matrigel and injected into the right shoulder of the mice, which were anesthetized with a xylazine (10 mg/kg) and ketamine (100 mg/kg) mixture by intraperitoneal injection. - Radiation: The sh-NC+R and sh-IDH2+R groups were given 2 Gy radiation at 3, 5, and 7 days after injection. Mice were anesthetized with a xylazine and ketamine mixture and radiated by X-RAD 225 OptiMAX (Precision X-ray Inc., USA) - Measurement: Tumor volume (V) was measured every 3 days in three dimensions (a, b, c) and calculated according to the following formula: V=abc×0.52. - Xenograft collection: Mice in the sh-NC and sh-IDH2 groups were sacrificed at 30 days after injection, while the other mice were euthanized at 45 days after implantation. Mice were euthanized by cervical dislocation according to American Veterinary Medical Association (AVMA) guidelines for the euthanasia of animals. Specifically, the thumb and index finger are placed on either side of the neck at the base of the skull. With the other hand, the base of the tail is quickly pulled, causing separation of the cervical vertebrae from the skull. |
| Experimental animals | 6-week-old male BALB/c-nu mice, weight 18-23g. |
| Housing and husbandry | - Housing: specific pathogen free; plastic and sterile cage; max 5 animals/cage; high adsorbing bedding material without dust, changed every week. - Husbandry conditions: automatic systems of temperature, humidity and light regulation (temperature: 20-24°C; humidity: 60±5%; dark/light cycle: 12/12h); water and food: autoclave sterilization. - Welfare-related assessments were conducted by two lab technicians. |
| Sample size | - 5 mice per group. - Sample size was calculated using PASS software (two-sample T-tests allowing unequal variance). |
| Allocating animals to  experimental groups | Mice were assigned to 4 experimental groups after randomization. |
| Experimental outcomes | To investigate the role of IDH2 in radiosensitivity of esophageal squamous cell carcinoma. |
| Statistical methods | Statistical analyses were performed with a two-tailed unpaired t-test. The differences were considered significant at *P*<0.05. |
